# P. M. B. A. of Texas

**Published:** 1889-02

**Authors:** 


### P. M. B. A. OF TEXAS.

In the list of those who paid assessment No. 8, published in our last issue, the names of Drs. J. D. Beek, J. T. Musick, J. S. Bruce and W. T. Richmond should have appeared. The last two names were accidentally omitted by the printer, and the assessments of the first two were not received until after the list had been closed.

Mrs. Tompkins received $108 net. Below is her receipt for the money:

$108. HOUSTON, TEX., January, 1889.

Received of Drs. W. A. Morris and F. E. Daniel, President and Secretary and Treasurer Physicians' Mutual Benefit Association of Texas, one hundred and eight dollars, the net amount collected under assessment No. 8.

MRS. IDA M. TOMPKINS.

Assessments 9, 10 and 11, for the benefit of the families of Drs. W. F. Buck, J. T. Y. Jameson and L. T. Maddox, were sent out on the 12th instant. The order is about holding its own.

THE NORTH AMERICAN PRACTITIONER is the name of the latest addition to the list of medical journals. It is published by Chas. Truax & Co., Chicago, monthly; at $1 a year, and is edited by Dr. Bayard Holmes. Vol. 1, No. 1, is on our desk.

